# ﻿Description of *Alvaniawangi* Xu, Qi & Kong, sp. nov. (Mollusca, Gastropoda, Littorinimorpha, Rissoidae) from the East China Sea

**DOI:** 10.3897/zookeys.1110.82173

**Published:** 2022-07-06

**Authors:** Biyang Xu, Lu Qi, Lingfeng Kong, Qi Li

**Affiliations:** 1 Key Laboratory of Mariculture, Ministry of Education, Ocean University of China, Qingdao, 266003, China Ocean University of China Qingdao China; 2 Laboratory for Marine Fisheries Science and Food Production Processes, Qingdao National Laboratory for Marine Science and Technology, Qingdao, 266237, China Laboratory for Marine Fisheries Science and Food Production Processes, Qingdao National Laboratory for Marine Science and Technology Qingdao China

**Keywords:** Micromolluscs, morphology, new species, phylogenetics, systematics

## Abstract

*Alvaniawangi* Xu, Qi & Kong, **sp. nov.** (Mollusca, Gastropoda, Littorinimorpha, Rissoidae) was discovered within the intertidal zone in the Nanji Islands and Zhoushan Islands, Zhejiang Province, China. It has a radula characteristic of *Alvania* Risso, 1826, a protoconch sculptured with micro pits and lamellae between spiral lirae, and a teleoconch with growth lines and subobsolete cords. Specimens were examined using an integrative taxonomic approach incorporating morphological observations and phylogenetic analyses of concatenated mitochondrial 16S rRNA and nuclear 28S rRNA gene sequences. The findings suggest that the new species is sister to *Alvaniacircinata* A. Adams, 1861 and is probably endemic to the shallow waters of the East China Sea.

## ﻿Introduction

Rissoidae Gray, 1847 is a family of highly diversified and widespread microgastropods (Ponder 1985; Hasegawa 2014; Criscione et al. 2017). Comprising hundreds of species, *Alvania* is one of the most diverse genera within Rissoidae. It is found worldwide, except in the Antarctic and sub-Antarctic regions (Ponder 1985), and in shallow to bathyal waters (e.g., Ávila et al. 2012; Hasegawa 2014; Hoffman and Freiwald 2021). *Alvania* is especially abundant in the Mediterranean Sea (Ávila et al. 2012; Criscione et al. 2017) and the North Atlantic (e.g., Warén 1973, 1974), and many new species from these regions have been described in the last five years (Amati and Chiarelli 2017; Bitlis and Öztürk 2017; Villari 2017; Amati et al. 2018, 2019, 2020a, b; Hoffman and Freiwald 2021). The Indo-West Pacific region (Ekman 1934, 1935, 1953) has the most diverse marine molluscan fauna (Lozouet and Plaziat 2008). However, only a few species of *Alvania* have been described or recorded from the Western Indian Ocean (e.g., Bozzetti 2017; Perugia 2021), Australasia (e.g., Laseron 1956; Ponder 1967), the Philippines (e.g., Poppe et al. 2018), Thailand (Bu-on and Dumgrongrojwattana 2019), China (e.g., Sowerby 1894; Zhang et al. 2016), and Japan (Adams 1861; Higo et al. 1999; Hasegawa 2014; Okutani 2017). Studies on *Alvania* from the East China Sea have been rarely reported. Only *Alvaniacircinata* A. Adams, 1861 was recorded from Kyushu (Okutani 2017); however, its type locality is Sado Island in the Sea of Japan (Adams 1861).

Classification of *Alvania* species based on their external features is rendered difficult by considerable convergence in shell characteristics and variations in the degree of development of the upper oviduct gland in females and the number of seminal receptacles in males (Johansson 1955; Fretter and Patil 1961; Golikov and Starobogatov 1975; Ponder 1985). Criscione et al. (2017) conducted the most comprehensive molecular phylogenetic study of *Alvania* to date, and provided a useful phylogenetic approach to better identify these rissoids. During two field studies conducted by the Laboratory of Shellfish Genetics and Breeding (hereafter LSGB) in 2020–2021, microgastropod samples were collected from the Nanji Islands and Zhoushan Islands, China. By integrating morphological observations and phylogenetic analyses based on the mitochondrial 16S rRNA gene fragment (hereafter 16S) and partial nuclear 28S rRNA gene (hereafter 28S), several rissoiform gastropods with peculiar shell characteristics were identified as belonging to a new species, described herein as *Alvaniawangi* Xu, Qi & Kong, sp. nov.

## ﻿Methods

**Figure 1.**
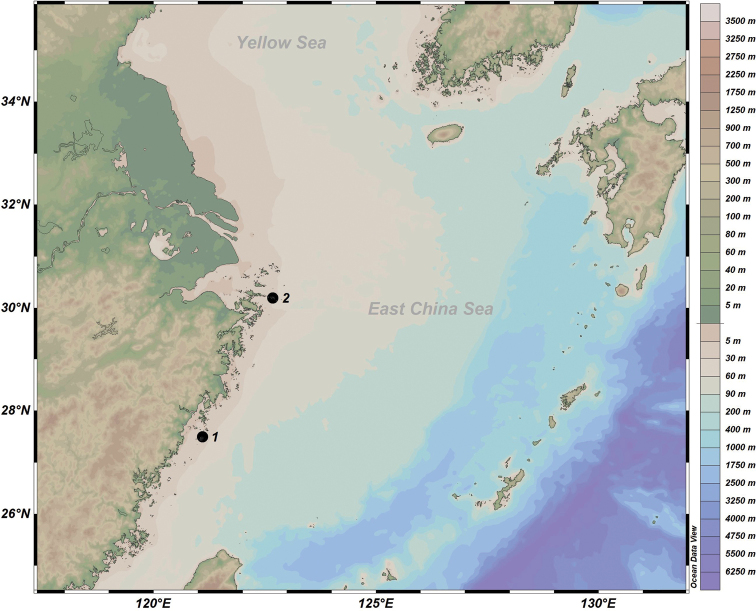
Map of sampling sites.

**Figure 2.**
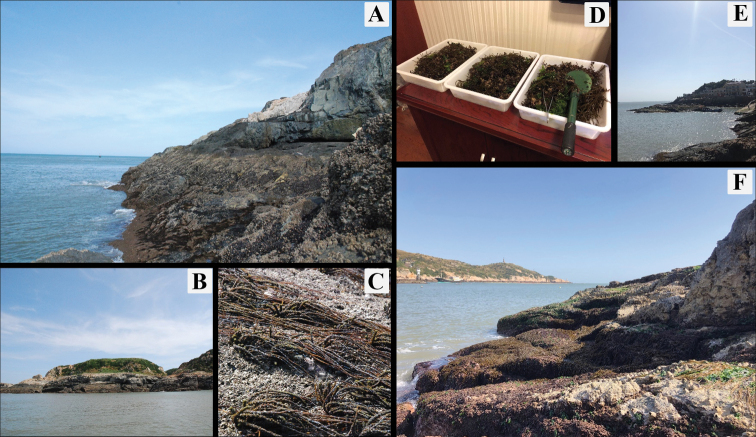
Pictures of the two sampling sites **A** rocky intertidal zonation on the coast of Dalei Island (location 1) **B** distant view of part of Dalei Island **C** algae growing on the lower intertidal zone of Dalei Island **D** algae collected from Miaozihu Island **E** distant view of part of Miaozihu Island **F** rocky intertidal zonation on the coast of Miaozihu Island (location 2) (photographs by Biyang Xu).

 Algae were scraped from intertidal rocks at Dalei Island and Miaozihu Island (Table 1; Figs 1, 2) in the East China Sea. The collected algae were washed manually and vigorously with seawater to obtain specimens of micromolluscs. The specimens were then subjected to the boiling method (Fukuda et al. 2007), fixed in 95% ethanol, and stored at –30 °C. Specimens mixed with debris were segregated, observed under a stereomicroscope (Nikon SMZ 800N; Nikon, Tokyo, Japan), and picked out using fine-tip forceps. Well-preserved specimens were then placed into 1.5 mL cryogenic vials containing 95% ethanol. The specimens were sonicated at a frequency of 40kHz for 2 minutes and selected as the type material on which further analysis and the new species description were based.

**Table 1.** Sampling information.

Location	Locality name	Collection date	Collector	Geo-coordinates
1	Dalei Island, Nanji Islands National Nature Reserve, Wenzhou, China	23 Jul. 2020	Biyang Xu, Lu Qi	27°29.82'N, 121°06.17'E
2	Miaozihu Island, Zhongjieshan Islands Special Marine Reserve, Zhoushan, China	09 Apr. 2021	Biyang Xu, Lu Qi	30°11.77'N, 122°41.41'E

Shells were photographed with a DS-Fi2 digital camera (Nikon, Tokyo, Japan) mounted on a stereomicroscope. The image stacks of standard views of the shells (Callomon 2019) were produced and combined using Helicon Focus 8.0.2. For scanning electron microscopy (SEM) studies, the radula was collected during DNA extraction following the method described by Qi et al. (2020), cleaned using 10% NaOH for 0.5 h, and rinsed in double-distilled water (ddH_2_O). The shells, opercula, and radulae were then gilded and examined using a Tescan Vega3 scanning electron microscope (Tescan, Brno, Czech Republic). The sampling locations were mapped using Ocean Data View 5.5.2 (Schlitzer 2021).

Genomic DNA was extracted from the specimens using the TIANamp Marine Animals DNA Kit (Tiangen, Beijing, China), following the manufacturer’s protocol. DNA was extracted in elution buffer (25 μL) and stored at 4 °C for short-term use. The 28S and 16S sequences were then amplified (Table 2). These gene markers have been widely and effectively used for phylogenetic studies of the family Rissoidae (Hausdorf et al. 2003; Criscione and Ponder 2013; Takano and Kano 2014; Baptista 2017; Criscione et al. 2017; Baptista et al. 2019). The target gene sequences were amplified using PCR; each sample (10 μL) contained 4 μL of DNA extract and 6 μL of PCR mix (0.2 μL ddH_2_O, 5 μL of 2× Taq Plus Master Mix II (Dye Plus; Vazyme, Nanjing, China), 0.4 μL of 10 μM forward primer, and 0.4 μL of 10 μM reverse primer). The amplified products were verified using 2% (w/v) agarose gel electrophoresis. After running the gel at 115V for 0.5 h, the products were stained with ethidium bromide, visualized using a UV transilluminator (Peiqing, Shanghai, China), and, finally, Sanger sequenced using the PCR primer pairs (Table 2). The sequences obtained were manually corrected for misreads, and the forward and reverse strands were both primer-trimmed and merged into contigs using SeqMan v.6 (DNASTAR, Madison, WI, USA). The assembled 28S and 16S sequences were BLAST searched to check for contamination and then deposited in GenBank (for accession numbers, see Suppl. material 1). The 28S and 16S sequences of related Rissoidae species were retrieved from GenBank (Suppl. material 1). *Amphithalamusfulcira* (Laseron, 1956) (family Anabathridae) was selected as the outgroup.

**Table 2.** Target gene, primer details and PCR conditions (temperature, time, and number of cycles) applied in the present study.

Gene	Primer	Sequence 5’-3’	Reference	PCR conditions
28S	28SDKF	F: GATCGGACGAGATTACCCGCTGAA	Strong et al. 2011	94 °C (7’), 58 °C (1’), 72 °C (2’) [x1]; 94 °C (1’), 52 °C (1’), 72 °C (2’) [x35]; 94 °C (1’), 52 °C (1’), 72 °C (7’) [x1]
	LSU 1600R	R: AGCGCCATCCATTTTCAGG	Williams et al. 2003	
16S	16SARis	F: TGCCTGTTTAGCAAAAACAT	Criscione and Ponder 2013	94 °C (5’), 52 °C (30’’), 72 °C (1’) [x1]; 94 °C (30’’), 52 °C (30’’), 72 °C (1’) [x40]; 94 °C (30’’), 52 °C (30’’), 72 °C (7’) [x1]
	16SBRis	R: CCGGTCTGAACTCAGATCATGT		

The 16S and 28S sequences were aligned independently using MUSCLE v3.8.31 (Edgar 2004). Areas of uncertain alignment were removed using Gblocks 0.91b (Castresana 2000) with the parameters -t=d -b1=36 -b2=45 -b3=3 -b4=5 -b5=a and -t=d -b1=25 -b2=41 -b3=8 -b4=10 -b5=a for 16S and 28S sequences, respectively. Substitution saturation of 16S and 28S was tested using DAMBE 7 (Xia 2018). The aligned 16S and 28S sequences from the same species were concatenated into one sequence with PhyloSuite 1.2.2 (Zhang et al. 2020) for two-gene analysis. Phylogenetic analyses were conducted for concatenated sequences using maximum likelihood (ML) and Bayesian inference (BI) methods. Prior to the phylogenetic analyses, the Bayesian information criterion (Schwarz 1978) was calculated using jModelTest 2.1.10 (Guindon and Gascuel 2003; Darriba et al. 2012) and GTR + G + I was identified as the best-fit model of nucleotide substitution. ML analyses were performed using RAxML v8.2.12 (Stamatakis 2014) and node support was assessed using 1000 ML bootstrap replicates. BI analyses were conducted using MrBayes v3.2.3 (Ronquist et al. 2012). Bayesian posterior probabilities were estimated by running 10,000,000 generations of four Markov chain Monte Carlo chains, including one cold chain and three heated chains, in two parallel runs. Trees were sampled every 1000 generations. The initial 25% of the trees was discarded as burn-in, and the remaining trees were summarized as 50% majority-rule trees. Stationarity was reached when the average standard deviation of split frequencies (Ronquist et al. 2012) was less than 0.01 and the potential scale reduction factor (Gelman and Rubin 1992) approached 1.0. Trees were graphed using FigTree v1.4.4 (Rambaut 2018). Pairwise distances of 16S (Table 3) and 28S (Table 4) within the *Alvania* clade were computed with MEGA X (Kumar et al. 2018), using the substitution model K2P.

**Table 3.** K2P pairwise sequence distances (in percentage) between the analyzed specimens based on 16S rRNA.

Species	1	2	3	4	5	6	7	8	9	10	11	12
*Alvaniawangi* sp. nov.	–											
*Alvaniaaeoliae*	12.93	–										
*Alvaniacircinata*	8.69	13.37	–									
*Alvaniadiscors*	13.19	7.16	13.40	–								
*Alvanialanciae*	13.46	6.00	14.20	8.36	–							
*Alvanialineata*	12.93	0.00	13.37	7.16	6.00	–						
*Alvanianovarensis*	6.78	11.83	8.64	12.59	12.84	11.83	–					
*Alvaniaogasawarana*	7.74	12.63	9.11	12.86	13.66	12.63	5.33	–				
*Alvaniascabra*	14.83	8.21	15.27	9.39	8.92	8.21	13.40	14.47	–			
*Alvaniatenera*	15.58	14.26	18.33	14.13	12.31	14.26	14.20	15.02	13.97	–		
*Crisillagalvagni*	13.78	12.19	15.24	13.73	11.96	12.19	13.66	12.89	12.75	14.55	–	
*Cingulatrifasciata*	13.90	10.77	14.14	12.26	11.25	10.77	12.29	13.65	12.32	12.35	12.60	–

All the materials analyzed in this study are deposited in the Laboratory of Shellfish Genetics and Breeding, Fisheries College, Ocean University of China, Qingdao, China.

**Table 4.** K2P pairwise sequence distances (in percentage) between the analyzed specimens based on 28S rRNA.

Species	1	2	3	4	5	6	7	8	9	10	11	12
*Alvaniawangi* sp. nov.	–											
*Alvaniaaeoliae*	4.51	–										
*Alvaniacircinata*	1.14	5.25	–									
*Alvaniadiscors*	5.89	2.69	6.06	–								
*Alvanialanciae*	4.67	1.29	5.32	2.30	–							
*Alvanialineata*	4.51	0.00	5.25	2.69	1.29	–						
*Alvanianovarensis*	3.22	5.56	3.38	6.29	5.47	5.56	–					
*Alvaniaogasawarana*	3.15	5.39	3.15	6.13	5.23	5.39	1.44	–				
*Alvaniascabra*	5.00	1.29	5.73	3.56	1.75	1.29	5.96	5.80	–			
*Alvaniatenera*	4.43	2.45	5.08	3.55	2.53	2.45	5.38	5.14	3.15	–		
*Crisillagalvagni*	4.75	2.37	5.24	3.79	2.53	2.37	5.71	5.38	3.00	2.60	–	
*Cingulatrifasciata*	4.27	3.63	4.67	4.43	3.23	3.63	5.30	5.06	3.87	2.69	2.85	–

## ﻿Results

The 16S (489 bp) and 28S (1414 bp) regions of *Alvaniawangi* Xu, Qi & Kong, sp. nov. were successfully amplified and sequenced. No cross-contamination or substitution saturation of 16S or 28S was detected. The K2P distances between *Alvaniawangi* Xu, Qi & Kong, sp. nov. and the analyzed species ranged from 6.78% to 15.58% and 1.14% to 5.89% for 16S and 28S, respectively (Tables 3, 4). *Alvaniawangi* Xu, Qi & Kong, sp. nov. is closely related to other *Alvania* species. BI (Fig. 5) and ML (Fig. 6) phylogenetic trees indicated that it is placed in the *Alvania* clade and sister to *Alvaniacircinata* A. Adams, 1861, with 100% nodal support from both BI and ML methods.

### ﻿Systematics

**Family Rissoidae Gray, 1847**

#### Genus *Alvania* Risso, 1826

##### ﻿ Alvania wangi

Taxon classificationAnimaliaLittorinimorphaRissoidae

Xu, Qi & Kong sp. nov.

F5345B14-0E3B-5ECF-AE7D-85CBCBAE7C51

https://zoobank.org/680409A3-C0A8-4571-98E5-3B28D80A7C86

Figs 3 , 4

###### Type locality.

China, Zhejiang: Pingyang County, the Nanji Islands National Nature Reserve, Dalei Island, 27°29.82'N, 121°06.17'E.

***Holotype***: Alcohol-fixed, photographed by SEM; original label: “CN, ZJ, Pingyang, Dalei, 27°29.82'N, 121°06.17'E, 23 Jul. 2020, B.Y. Xu & L. Qi” “LSGB mg325408 0601”.

***Paratypes***: Alcohol-fixed, five specimens, original label: “CN, ZJ, Pingyang, Dalei, 27°29.82'N, 121°06.17'E, 23 Jul. 2020, B.Y. Xu & L. Qi” “LSGB mg325408 0602 to 0606”; alcohol-fixed, ten specimens, original label: “CN, ZJ, Zhoushan, Miaozihu, 30°11.77'N, 122°41.41'E, 09 Apr. 2021, B.Y. Xu & L. Qi” “LSGB mg316141 0601 to 0610”.

###### Diagnosis.

Shell minute, ovate-conical, thin, with weakly convex whorls, non-umbilicate. Protoconch paucispiral, sculptured with micro pits and lamellae between spiral lirae. Teleoconch with subobsolete cords and growth lines. Umbilicus chink very narrow and slit-like. Aperture oval, broadly rounded anteriorly, slightly angled posteriorly; peristome simple; outer lip orthocline, without varix. Periostracum thin.

###### Description.

**Figure 3.**
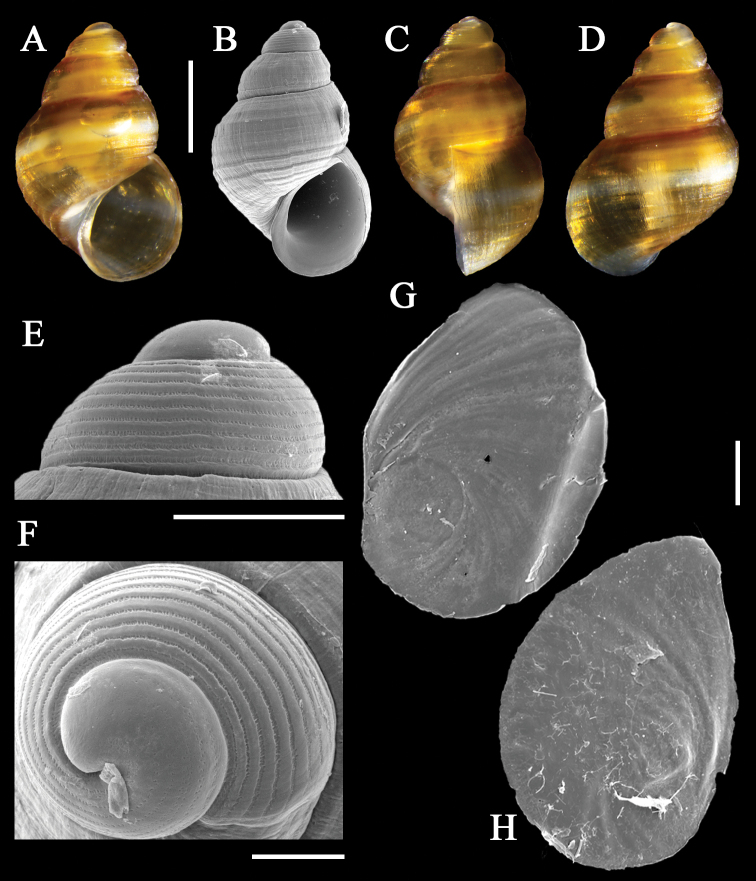
Holotype of *Alvaniawangi* Xu, Qi & Kong, sp. nov. (**A–D**) shell **A** apertural view of shell **B** scanning electron micrographs of apertural view of shell **C** lateral view of shell **D** dorsal view of shell **E** protoconch **F** apical view of protoconch (**G, H)** operculum **G** outer face of operculum **H** inner face of operculum. Scale bars: 500 μm (**A–D**); 200 μm (**E**); 100 μm (**F–H**).

**Figure 4.**
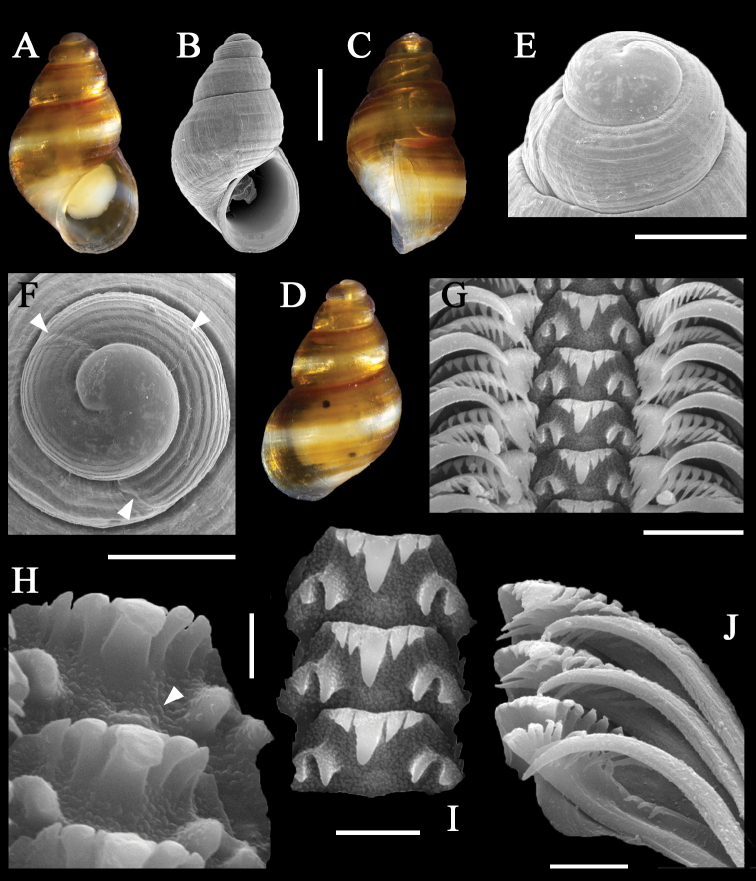
Paratype of *Alvaniawangi* Xu, Qi & Kong, sp. nov. (**A–D)** shell **A** apertural view of shell **B** scanning electron micrographs of apertural view of shell **C** lateral view of shell **D** dorsal view of shell **E** protoconch **F** apical view of protoconch (top two arrowheads show the two growth lines of the protoconch; bottom arrowhead indicates demarcation between protoconch and teleoconch) **G** radula **H** oblique view of central teeth (arrowhead indicates pustules on base of central teeth) **I** central teeth **J** lateral and marginal teeth. Scale bars: 500 μm (**A–D**); 200 μm (**E, F**); 10 μm (**G**); 2 μm (**H**); 5 μm (**I, J**).

***Shell***: (Figs 3A–D, 4A–D) minute, ovate-conical, semitransparent, thin but not fragile. Protoconch (Figs 3E, F, 4E, F) dome-shaped, with 1.5 whorls, height ~190 μm, diameter 353 μm, translucent; nucleus apparently smooth, followed by half whorl ornamented with dense and irregular micro pits (Fig. 3F) which subsequently fuse into micro-lamellae between 9–10 spiral lirae (Fig. 3E, F), roughly three times wider than interspaces. Protoconch-teleoconch border simple, marked by a shallow depression of the spire (Fig. 4F). Teleoconch with 2 whorls, slightly convex, with 3–4 subobsolete spiral cords on penultimate whorl, 9–10 on body whorl, 3–4 relatively distinct spiral cords on base, roughly equidistant and broader than interspaces, crossing with exceedingly fine growth lines, and few weak spiral furrows in the periumbilical area. Periphery of body whorl broadly rounded. Suture impressed, simple. Aperture oval, with simple peristome, angled posteriorly, rounded anteriorly; inner lip narrow, anteriorly slightly separated from lower base, posteriorly attached to base; outer lip orthocline, without external varix and internal lirae. Umbilical chink represented by a narrow and short groove. Periostracum very thin, barely visible. Background color yellowish and brownish, forming interlaced band.

***Operculum***: (Fig. 3G, H) subovate, horny, simple, thin, smooth, posteriorly broadly angled, anteriorly rounded; nucleus eccentric; last whorl long and large, yellowish, and translucent.

***Radula***: (Fig. 4G–J) typical of *Alvania*. Central teeth $\frac{2-3+1+2-3}{1+11+1}$, with long triangular cutting edge, small cusps, a single pair of basal denticles and a pair of smaller denticles produced from the thickened lateral margins; U-shaped ventral extension poorly to not developed. Lateral teeth 3 + 1 + 6–7, elongate, each with triangular and asymmetric cusps: larger primary cusp long and wide; 3 inner and 6–7 outer, smaller pointed denticles at the sides. Marginal teeth elongated, cusps subequal; inner marginal teeth with ~17 cusps on outer 1/3 of teeth, outer marginal teeth with ~6–7 cusps on inner distal 1/3.

***Soft parts***: Yellowish head and foot. A pair of black-pigmented eyes (Fig. 4D) can be seen through the translucent shell. Cephalic tentacles yellow, behind the eyes.

###### Etymology.

The species is named after Prof. Rucai Wang, who established LSGB and was one of the founders of shellfish culture in China.

###### Known distribution.

In addition to the type locality, this species can also be found in the middle intertidal zone of Miaozihu Island, the northeastern part of Zhoushan City, China, 30°11.77'N, 122°41.41'E.

###### Remarks.

The characteristics of *Alvaniawangi* Xu, Qi & Kong, sp. nov. are consistent with those of *Alvania* described by Risso (1826) and Ponder (1967, 1985). It also possesses some unique features, such as the subobsolete spiral cords and the protoconch sculptured with pits, lirae, and micro-lamellae. The new species can be clearly distinguished from other *Alvania* species in adjacent waters (Suppl. material 2). Additionally, it resembles *A.carinata* (da Costa, 1778), *A.cimex* (Linnaeus, 1758), *A.lineata* Risso, 1826, *A.punctura* (Montagu, 1803), and *A.scabra* (Philippi, 1844) in radula morphology, but differs both in clathrate sculpture and protoconch features. Among all species of Rissoidae, the new species may be most closely related to *Crisillasimulans* (Locard, 1886) and *C.perminima* (Manzoni, 1868), which are found independently in the Mediterranean Sea (Morena and Luigi 2005) and northwestern Africa (Oliver et al. 2019). It shares similar color patterns, sizes, the general outline of the shell, and the lack of conspicuous axial sculpture with the two *Crisilla* species. However, it differs from these species in the presence of 9–10 relatively clear spiral cords on the body whorl and in the spiral microsculptures of the protoconch. Furthermore, the new species has a similar protoconch sculpture to those of *Cingulaaequa* (E. A. Smith, 1890) and *C.farquhari* (E. A. Smith, 1910) in terms of the number and sculpture of spiral lirae with axial micro-lamellae. However, the two *Cingula* species lack the rows of pits and smooth zones found in the protoconch of *Alvaniawangi* Xu, Qi & Kong, sp. nov. (Fig. 3E, F). Moreover, the central tooth of the new species (Fig. 4G–I) is similar to that of *Cingulatrifasciata* (J. Adams, 1800), the type species of *Cingula* Fleming, 1818, which, however, has a moderately developed “U-shaped” ventral extension (Ponder 1985) and lacks pustules (Fig. 4H) on the base. *Alvaniawangi* Xu, Qi & Kong, sp. nov. can be distinguished from the above-mentioned species based on its peculiar characteristics and is therefore regarded as a distinct species of *Alvania*.

## ﻿Discussion

**Figure 5.**
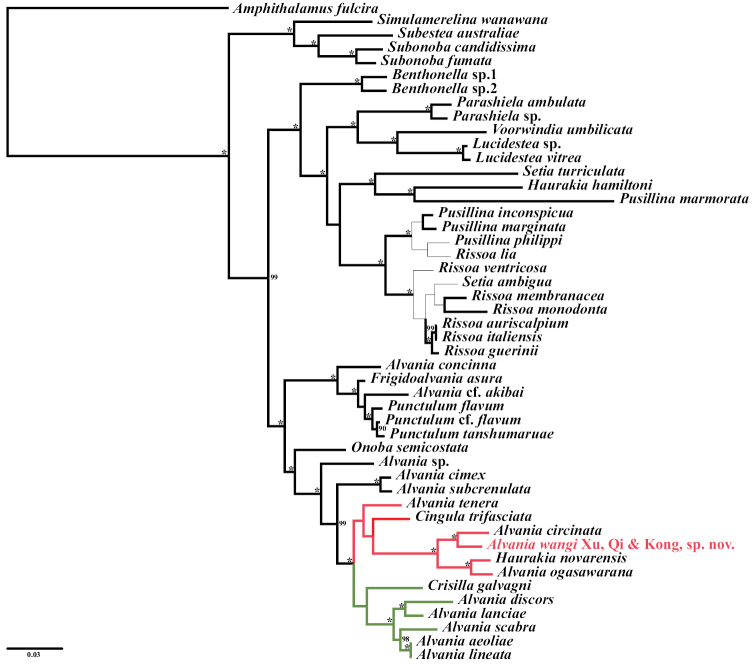
Bayesian consensus phylogram based on analysis of the concatenated 16S and 28S sequences. Numbers on branches indicate nodal support (in percentage) by Bayesian posterior probabilities (BPP; only values ≥ 90% are shown; values of 100% are represented by asterisks). Thick lines mark branches that are consistent with the topology of the ML tree.

**Figure 6.**
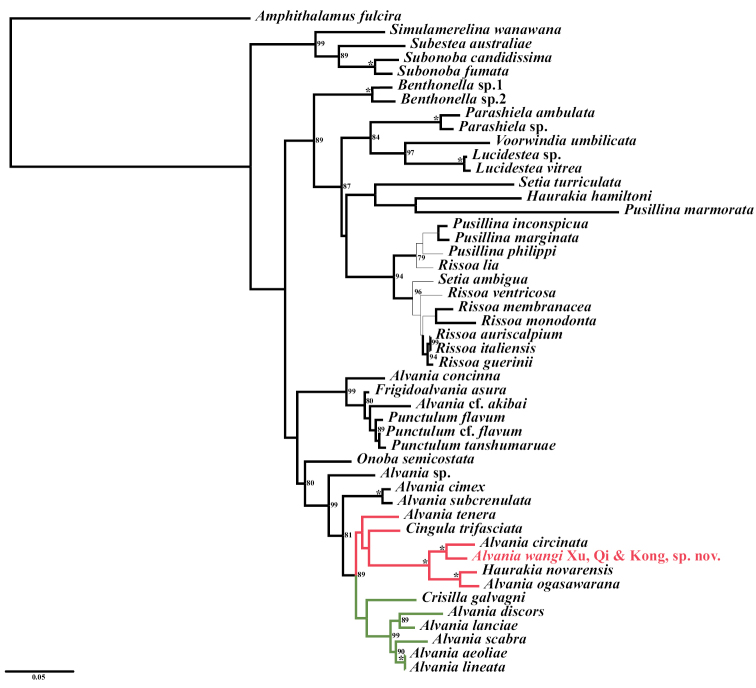
Maximum-likelihood phylogram based on analysis of the concatenated 16S and 28S sequences. Numbers on branches indicate nodal support (in percentage) by ML bootstrap (BTSP; only values ≥ 70% are shown; values of 100% are represented by asterisks). Thick lines mark branches that are consistent with the topology of the BI tree.

The new species described in the present study differs from the previously reported *Alvania* species with respect to the sculptures on its shells. Molecular evidence supports the morphological identification. Moreover, phylogenetic reconstruction revealed consistent topologies of *Alvania* in both BI (Fig. 5) and ML (Fig. 6) analyses, and confirmed that *Alvaniawangi* Xu, Qi & Kong, sp. nov. is a valid species within the *Alvania* lineage.

Notably, the addition of *Alvaniawangi* Xu, Qi & Kong, sp. nov. changes the topology of some subclades within the *Alvania* clade (Criscione et al. 2017). *Alvaniatenera* (Philippi, 1844) is sister to the clade that includes *Cingulatrifasciata* (J. Adams, 1800), *A.circinata* A. Adams, 1861, *Alvaniawangi* Xu, Qi & Kong, sp. nov., *A.ogasawarana* (Pilsbry, 1904), and *Haurakianovarensis* (Frauenfeld, 1867). The clade marked in green (Figs 5, 6) includes *Crisillagalvagni* (Aradas & Maggiore, 1844), *A.discors* (T. Brown, 1818), *A.lanciae* (Calcara, 1845), *A.scabra* (Philippi, 1844), *A.aeoliae* Palazzi, 1988, and *A.lineata* Risso, 1826. *Crisillagalvagni* did not cluster with *A.tenera* in the subclade marked in red (Figs 5, 6), although both species have an ovate-conical shell sculptured with spiral cords and weaker growth lines. Instead, *C.galvagni* is sister to the five *Alvania* species mentioned above, which have conical shells with strong axial ribs but weaker spirals. This indicates that *C.galvagni* is closely related to *Alvania* (Ponder 1985). However, the *Alvania* clade remains polyphyletic (Criscione et al. 2017), which is particularly reflected in the subclade marked in red (Figs 5, 6).

In the subclade marked in red (Figs 5, 6), *Alvaniawangi* Xu, Qi & Kong, sp. nov. shows a close relationship with *A.circinata* A. Adams, 1861, with 100% nodal support (Figs 5, 6). *Alvaniacircinata* was originally described from Sado Island (Adams 1861) in the Sea of Japan, and was later found in the intertidal zone of the Boso Peninsula, Oga Peninsula, and Kyushu (Okutani 2017). *Alvaniacircinata* is currently regarded as taxon inquirendum (Criscione et al. 2017) owing to inadequate descriptions, undesignated types, and scattered or even lost potential syntypes (Ponder and De Keyzer 1992; Hasegawa 2014). Thus, the new species described in the present study provides genetic support for reassigning *A.circinata* to *Alvania* and further clues for solving the taxonomic uncertainty surrounding this species.

The protoconch of *Alvaniawangi* Xu, Qi & Kong, sp. nov. is not sculptured with granules between a few spiral lirae like that of most *Alvania* species (Ponder 1985). It has rows of shallow pits and a smooth area in protoconch I and lamellae between the lirae in protoconch II (Figs 3E, F, 4E, F). This structure might be a remnant of the early stages of the evolution of *Alvania* (Ponder 1985). *Alvaniawangi* Xu, Qi & Kong, sp. nov. shows modifications of the general pattern of the protoconch and is probably a direct developer (Ponder 1985) with limited dispersal ability to achieve an extensive geographical distribution (Thorson 1950; Shuto 1974; Hansen 1980). Notably, it does not cluster with bathyal rissoid gastropods (Hasegawa 2014; Criscione et al. 2017) and possesses distinct black eyes, which are indicative of its shallow water origin (Hasegawa 2014). Moreover, this species has not been recorded in Japan (Takenori Sasaki, personal communication), and other species of *Alvania* in Japan are currently known to inhabit only the bathyal depths of the Sea of Japan and adjacent waters (Hasegawa 2014). Considering that this deep basin is a barrier for the dispersal of shallow water lecithotrophic species (Shuto 1974), *Alvaniawangi* Xu, Qi & Kong, sp. nov. may be endemic to the East China Sea.

## Supplementary Material

XML Treatment for Alvania wangi

## Supplementary materials

Supplementary material 1 GenBank accession numbers for species included in the molecular analyses Data type docx file Explanation note GenBank accession numbers for species included in the molecular analyses. https://binary.pensoft.net/file/711353This dataset is made available under the Open Database License
(http://opendatacommons.org/licenses/odbl/1.0/). The Open Database License (ODbL) is a
license agreement intended to allow users to freely share, modify, and use this
Dataset while maintaining this same freedom for others, provided that the original
source and author(s) are credited.Biyang Xu, Lu Qi, Lingfeng Kong, Qi Li

Supplementary material 2 A comparison among *Alvania* species found in the East China sea and adjacent waters Data type docx file Explanation note A comparison among *Alvania* species found in the East China sea and adjacent waters. https://binary.pensoft.net/file/711354This dataset is made available under the Open Database License
(http://opendatacommons.org/licenses/odbl/1.0/). The Open Database License (ODbL) is a
license agreement intended to allow users to freely share, modify, and use this
Dataset while maintaining this same freedom for others, provided that the original
source and author(s) are credited.Biyang Xu, Lu Qi, Lingfeng Kong, Qi Li
