# Supplementary material for: ﻿Description of Alvaniawangi Xu, Qi & Kong, sp. nov. (Mollusca, Gastropoda, Littorinimorpha, Rissoidae) from the East China Sea
Source: Zookeys. 2022 Jul 6;1110:201–17. doi: 10.3897/zookeys.1110.82173 (PMC10426732; doi:10.3897/zookeys.1110.82173)
Supplement: Supplementary material 1 — GenBank accession numbers for species included in the molecular analyses [file zookeys-1110-201_article-82173__-s001.docx]

**Suppl. file 1**

GenBank accession numbers for species included in the molecular analyses

| **Family** | **Species** | **28S AN** | **16S AN** | **Reference** |
| --- | --- | --- | --- | --- |
| Anabathridae Keen, 1971 | *Amphithalamus fulcira* (Laseron, 1956) | AB930345 | AB930417 | Takano and Kano 2014 |
| Rissoidae Gray, 1847 | *Alvania wangi* Xu, Qi & Kong, sp. nov. | OM295678 | OM295677 | This study |
|  | *Alvania aeoliae* Palazzi, 1988 | KR698251 | KR698192 | Criscione et al. 2017 |
|  | *Alvania* cf. *akibai* | KR698252 | KR698193 | Criscione et al. 2017 |
|  | *Alvania cimex* (Linnaeus, 1758) | KC109987 | KC109935 | Criscione and Ponder 2013 |
|  | *Alvania circinata* A. Adams, 1861 | KR698253 | KR698194 | Criscione et al. 2017 |
|  | *Alvania concinna* A. Adams, 1861 | KR698254 | KR698195 | Criscione et al. 2017 |
|  | *Alvania discors* (T. Brown, 1818) | KR698255 | KR698196 | Criscione et al. 2017 |
|  | *Alvania lanciae* (Calcara, 1845) | KR698257 | KR698198 | Criscione et al. 2017 |
|  | *Alvania lineata* Risso, 1826 | KR698258 | KR698199 | Criscione et al. 2017 |
|  | *Alvania ogasawarana* (Pilsbry, 1904) | KR698268 | KR698210 | Criscione et al. 2017 |
|  | *Alvania scabra* (Philippi, 1844) | KR698259 | KR698201 | Criscione et al. 2017 |
|  | *Alvania* sp. | KR698260 | KR698202 | Criscione et al. 2017 |
|  | *Alvania subcrenulata* (Bucquoy, Dautzenberg & Dollfus, 1884) | KR698261 | KR698203 | Criscione et al. 2017 |
|  | *Alvania tenera* (Philippi, 1844) | KR698262 | KR698204 | Criscione et al. 2017 |
|  | *Benthonella* sp.1 | KR698265 | KR698207 | Criscione et al. 2017 |
|  | *Benthonella* sp.2 | AB930363 | AB930422 | Takano and Kano 2014 |
|  | *Cingula trifasciata* (J. Adams, 1800) | KR698266 | KR698208 | Criscione et al. 2017 |
|  | *Crisilla galvagni* (Aradas & Maggiore, 1844) | KR698269 | KR698212 | Criscione et al. 2017 |
|  | *Frigidoalvania asura* (Yokoyama, 1926) | KR698270 | KR698213 | Criscione et al. 2017 |
|  | *Haurakia hamiltoni* (Suter, 1898) | KR698271 | KR698214 | Criscione et al. 2017 |
|  | *Haurakia marmorata* (Hedley, 1907) | KR698299 | KR698248 | Criscione et al. 2017 |
|  | *Haurakia novarensis* (Frauenfeld, 1867) | KR698267 | KR698209 | Criscione et al. 2017 |
|  | *Lucidestea* sp. | AB930347 | KR698215 | Takano and Kano 2014; Criscione et al. 2017 |
|  | *Lucidestea vitrea* Laseron, 1956 | KR698272 | KR698216 | Criscione et al. 2017 |
|  | *Onoba semicostata* (Montagu, 1803) | KR698277 | KR698223 | Criscione et al. 2017 |
|  | *Parashiela ambulata* Laseron, 1956 | KR698278 | KR698224 | Criscione et al. 2017 |
|  | *Parashiela* sp. | KR698279 | KR698225 | Criscione et al. 2017 |
|  | *Punctulum* cf. *flavum* | KR698281 | KR698227 | Criscione et al. 2017 |
|  | *Punctulum flavum* (Okutani, 1964) | KR698256 | KR698228 | Criscione et al. 2017 |
|  | *Punctulum tanshumaruae* Hasegawa, 2014 | KR698282 | KR698229 | Criscione et al. 2017 |
|  | *Pusillina inconspicua* (Alder, 1844) | KR698283 | KR698230 | Criscione et al. 2017 |
|  | *Pusillina marginata* (Michaud, 1830) | KR698284 | KR698231 | Criscione et al. 2017 |
|  | *Pusillina philippi* (Aradas & Maggiore, 1844) | KR698285 | KR698232 | Criscione et al. 2017 |
|  | *Rissoa auriscalpium* (Linnaeus, 1758) | KR698287 | KR698233 | Criscione et al. 2017 |
|  | *Rissoa guerinii* Récluz, 1843 | KR698288 | KR698234 | Criscione et al. 2017 |
|  | *Rissoa italiensis* Verduin, 1985 | KR698289 | KR698235 | Criscione et al. 2017 |
|  | *Rissoa lia* (Monterosato, 1884) | KR698291 | KR698237 | Criscione et al. 2017 |
|  | *Rissoa membranacea* (J. Adams, 1800) | KR698290 | KR698236 | Criscione et al. 2017 |
|  | *Rissoa monodonta* Philippi, 1836 | KR698292 | KR698238 | Criscione et al. 2017 |
|  | *Rissoa ventricosa* Desmarest, 1814 | KC110026 | KC109973 | Criscione and Ponder 2013 |
|  | *Setia ambigua* (Brugnone, 1873) | KR698296 | KR698244 | Criscione et al. 2017 |
|  | *Setia turriculata* Monterosato, 1884 | KR698297 | KR698245 | Criscione et al. 2017 |
|  | *Simulamerelina wanawana* (Kay, 1979) | KR698273 | KR698246 | Criscione et al. 2017 |
|  | *Subestea australiae*(Frauenfeld, 1867) | KC110031 | KC109978 | Criscione and Ponder 2013 |
|  | *Subonoba candidissima* (Webster, 1905) | KR698264 | KR698206 | Criscione et al. 2017 |
|  | *Subonoba fumata* (Suter, 1898) | KR698298 | KR698247 | Criscione et al. 2017 |
|  | *Voorwindia umbilicata* Ponder, 1985 | KR698300 | KR698249 | Criscione et al. 2017 |

**References**

Criscione F, Ponder WF (2013) A phylogenetic analysis of rissooidean and cingulopsoidean families (Gastropoda: Caenogastropoda). Molecular Phylogenetics and Evolution 66: 1075-1082. https://doi.org/10.1016/j.ympev.2012.11.026

Criscione F, Ponder WF, Köhler F, Takano T, Kano Y (2017) A molecular phylogeny of Rissoidae (Caenogastropoda: Rissooidea) allows testing the diagnostic utility of morphological traits. Zoological Journal of the Linnean Society 179: 23-40. https://doi.org/10.1111/zoj.12447

Takano T, Kano Y (2014) Molecular phylogenetic investigations of the relationships of the echinoderm-parasite family Eulimidae within Hypsogastropoda (Mollusca). Molecular Phylogenetics and Evolution 79: 258-269. https://doi.org/10.1016/j.ympev.2014.06.021
