# Supplementary material for: ﻿Description of Alvaniawangi Xu, Qi & Kong, sp. nov. (Mollusca, Gastropoda, Littorinimorpha, Rissoidae) from the East China Sea
Source: Zookeys. 2022 Jul 6;1110:201–17. doi: 10.3897/zookeys.1110.82173 (PMC10426732; doi:10.3897/zookeys.1110.82173)
Supplement: Supplementary material 2 — A comparison among Alvania species found in the East China sea and adjacent waters [file zookeys-1110-201_article-82173__-s002.docx]

**Suppl. file 2**

A comparison among *Alvania* species found in the East China sea and adjacent waters (“-”: no information available)

| **Species** | **Locality** | **Shell** | **Protoconch** | **Umbilicus** | **Aperture** | **Central teeth** | **Lateral teeth** | **Marginal teeth** | **Reference** |
| --- | --- | --- | --- | --- | --- | --- | --- | --- | --- |
| *Alvania wangi* Xu, Qi & Kong, sp. nov. | Nanji Islands, Zhoushan Islands, China | Shell with yellowish and brownish band, ovate-conic, sculptured with subobsolete cords and numerical axial growth lines | Dome-shaped, sculptured with micro pits and lamellae between the spiral lirae | Narrow | Ovate | 2–3+1+2–3 | 3 + 1 + 6–7 | Inner marginal teeth with 17 cusps,  outer marginal teeth with 6–7 cusps | This study |
| *Alvania concinna* | Hebei Province, Shandong Province, China; Sagami Bay, northern Kyushu, Japan; Kurile Islands | Shell reddish brown, elongate conical, sculptured with many spiral ribs and strong axial ribs | smooth and no ribs | Narrow | Broadly ovate | - | - | - | Zhang et al. 2016;  Okutani 2017 |
| *Alvania gradatoides* | Green Island, China | Shell white, conical, sculptured with slightly stronger axial ribs | Slightly depressed | Narrow | Ovate | - | - | - | Finlay 1930;  Huang and Lin 2012 |
| *Alvania isolata* | Green Island, China | Shell white with irregular brown patches, broadly conical, strongly clathrate-sculptured | Relatively large, sculptured with fine and spiral lirae | Imperforate | Broadly ovate | - | - | - | Laseron 1956;  Ponder 1985;  Huang and Lin 2012 |
| *Alvania maya* | Middle Yellow Sea；Sagami Bay, Noto Peninsula, Hokkaido, Japan | Shell light yellowish brown, elongate conical, sculptured with strong spiral ribs and fine axial lirae | - | Narrow | Ovate | - | - | - | Zhang et al 2016;  Okutani 2017 |
| *Alvania circinata* | Boso Peninsula, Oga Peninsula, Kyushu, Japan | Shell yellowish brown with irregular brown blotches, elongate conical, sculptured only with strong spiral ribs | - | Narrow | Ovate | - | - | - | Okutani 2017 |
| *Alvania ogasawarana* | Boso Peninsula, Yamagata Prefecture, Ogasawara Islands, Japan | Shell creamwhite with indistinct brown spots below suture, ovate conical, sculptured with many strong axial and spiral ribs | - | Imperforate | Ovate | - | - | - | Pilsbry 1904;  Okutani 2017 |
| *Alvania akibai* | endemic to the Sea of Japan | Shell rather translucently white, conical, sculptured with distinct spiral cords and weaker axial ribs | Dome-shaped and large, sculptured with peculiar pockmarked minute pits | Narrow | Ovate | 4–6+1+4–6 | 6–8+1+6–7 | Inner marginal teeth with 6–8 cusps,  outer marginal teeth with 4 cusps | Hasegawa 2014;  Okutani 2017 |
| *Alvania* cf. *awa* | Niigata Prefecture, Japan | Shell white and covered by light brownish periostracum, elongate conical, sculptured with distinct spiral cords but obscure axial ribs | - | Narrow | - | - | - | - | Hasegawa 2014;  Okutani 2017 |
| *Alvania yamatoensis* | Yamato Bank, Japan | Shell opaquely white, elongate conical, sculpture weak axial and spiral ribs | Dome-shaped and relatively small, sculptured with wavy spiral riblets and minute raised dots | Imperforate or narrow | Evenly rounded | 5–6+1+5–6 | 7–8+1+6–8 | Inner marginal teeth with more than 10 cusps,  outer marginal teeth with 4 cusps | Hasegawa 2014;  Okutani 2017 |
| *Alvania nihonkaiensis* | Sea of Japan; Okhotsk Sea | Shell opaquely milk-white, conical, sculptured with spiral cords and regularly arranged axial ribs | Dome-shaped and relatively small, sculptured with peculiar zigzag lines | Narrow | Ovate | 5+1+5 | 5+1+5 | Inner marginal teeth with 4 cusps,  outer marginal teeth with 3 cusps | Hasegawa 2014;  Okutani 2017 |

**References**

Finlay HJ (1930) Additions to the Recent fauna of New Zealand. No. 3. Transactions of the New Zealand Institute 61: 222-247.

Hasegawa K (2014) A review of bathyal Rissoidae in the Sea of Japan and adjacent waters (Gastropoda: Rissooidea). In: Fujita T (Ed) Deep-sea fauna of the sea of Japan. National Museum of Nature and Science, Tokyo, 75-148.

Huang Z, Lin M (2012) The Living Species and Their Illlustrations in China's Seas (Part I) THE LIVING SPECIES IN CHINA'S SEAS. CHINA OCEAN PRESS, Beijing, 632 pp.

Laseron C (1956) The families Rissoinidae and Rissoidae (Mollusca) from the Solanderian and Dampierian zoogeographical provinces. Australian Journal of Marine and Freshwater Research 7: 384-484. https://doi.org/10.1071/MF9560384

Okutani T (2017) Marine Mollusks in Japan. Second Edition. Tokai University Press, Japan, 1375 pp.

Pilsbry HA (1904) New Japanese marine Mollusca: Gastropoda. Proceedings of the Academy of Natural Sciences of Philadelphia 56: 26-27.

Ponder WF (1985) A review of the genera of the Rissoidae (Mollusca: Mesogastropoda: Rissoacea). Records of the Australian Museum, Supplement 4: 1–221. https://doi.org/10.3853/j.0812-7387.4.1985.100

Zhang SP, Zhang JL, Chen ZY, Xu FS (2016) Mollusks of the Yellow Sea and Bohai Sea. Science Press, Beijing, 421 pp.
